# Effect of coil orientation on strength–duration time constant and I-wave activation with controllable pulse parameter transcranial magnetic stimulation

**DOI:** 10.1016/j.clinph.2015.05.017

**Published:** 2016-01

**Authors:** Kevin D’Ostilio, Stefan M. Goetz, Ricci Hannah, Matteo Ciocca, Raffaella Chieffo, Jui-Cheng A. Chen, Angel V. Peterchev, John C. Rothwell

**Affiliations:** a Sobell Department of Motor Neuroscience and Movement Disorders, UCL Institute of Neurology, London, UK; b MoVeRe Group, Cyclotron Research Centre, University of Liege, Belgium; c Department of Psychiatry and Behavioral Sciences, Duke University, Durham, NC, USA; d Technical University Munich, Munich, Germany; e Department of Neurological Science, University of Milan, Milan, Italy; f Department of Neurology, Scientific Institute Hospital San Raffaele, Milan, Italy; g Neuroscience Laboratory, Department of Neurology, China Medical University Hospital, Taichung, Taiwan; h School of Medicine, China Medical University, Taichung, Taiwan; i Department of Biomedical Engineering, Department of Electrical and Computer Engineering, Duke University, Durham, NC, USA

**Keywords:** Transcranial magnetic stimulation, Pulse width, Motor threshold, Strength–duration time constant, Input–output curve, Current direction

## Abstract

**Objective:**

To compare the strength–duration (S–D) time constants of motor cortex structures activated by current pulses oriented posterior–anterior (PA) or anterior–posterior (AP) across the central sulcus.

**Methods:**

Motor threshold and input–output curve, along with motor evoked potential (MEP) latencies, of first dorsal interosseus were determined at pulse widths of 30, 60, and 120 μs using a controllable pulse parameter (cTMS) device, with the coil oriented PA or AP. These were used to estimate the S–D time constant and we compared with data for responses evoked by cTMS of the ulnar nerve at the elbow.

**Results:**

The S–D time constant with PA was shorter than for AP stimulation (230.9 ± 97.2 vs. 294.2 ± 90.9 μs; *p* < 0.001). These values were similar to those calculated after stimulation of ulnar nerve (197 ± 47 μs). MEP latencies to AP, but not PA stimulation were affected by pulse width, showing longer latencies following short duration stimuli.

**Conclusion:**

PA and AP stimuli appear to activate the axons of neurons with different time constants. Short duration AP pulses are more selective than longer pulses in recruiting longer latency corticospinal output.

**Significance:**

More selective stimulation of neural elements may be achieved by manipulating pulse width and orientation.

## Highlights

• S–D time constants are longer for anterior–posterior than posterior–anterior induced currents.
• Brief (30 μs) anterior-posterior currents evoke the longest latency MEP.
• Selective stimulation of neural elements may be achieved by manipulating pulse width and orientation.

## 1 Introduction

Transcranial magnetic stimulation (TMS) is a non-invasive technique that has been widely used to investigate the physiology of the cerebral cortex. Most studies have been performed on the hand area of the primary motor area. Here, a single TMS pulse activates excitatory synaptic inputs to corticospinal neurons, initiating descending activity in the corticospinal tract and contraction of muscles on the opposite side of the body (Day et al., 1989). The physiological effects of the stimulus are usually quantified in terms of motor threshold, the intensity required to evoke a liminal EMG response in a target muscle (motor evoked potential; MEP), and input–output (I/O) curves, which measure how the response amplitude changes with stimulus intensity. Such measures are affected by CNS damage and disease, and associated pharmacological treatments. For example, antiepileptic drugs, such as carbamazepine, increase the threshold for stimulation because of their action on Na+ channels in nerve membrane (Ziemann, 2013); damage to the corticospinal system, such as after stroke, was reported to reduce the slope of the I/O relationship (Swayne et al., 2008).

However, until recently it was difficult to measure another parameter that is often used to characterize the excitability of peripheral nerve: the strength–duration (S–D) time constant, which describes how the threshold for stimulation depends on a combination of the duration and intensity of the stimulus pulse. Its value depends on the electrical capacitance of the membrane as well as the resistances and dynamics of the ion channels within it (Boinagrov et al., 2010, Bostock, 1983, Geddes, 2004, Rattay et al., 2012). In peripheral nerves, the S–D time constant is known to change in a variety of conditions that affect the membrane such as ALS and CIDP (Lin et al., 2011, Vucic and Kiernan, 2006). Most commercially available TMS machines do not allow the duration of the stimulus pulse to be modified, and therefore cannot estimate central S–D time constants in motor cortex. Recently, Peterchev and colleagues developed a new device in which it is possible to change the duration of the TMS output over a limited range. They calculated values for activation of motor cortex in healthy volunteers (Peterchev et al., 2013) that was in agreement with an earlier estimate of Barker and colleagues who used a different technological approach (Barker et al., 1991). Both were similar to the S–D time constant for magnetic stimulation of peripheral nerve estimated by Barker et al. (1991), and hence compatible with the idea that at threshold, TMS of motor cortex likely activates relatively large myelinated axons in brain.

Peterchev et al. (2013) used monophasic TMS pulses which induced posterior–anterior (PA) current flow approximately perpendicular to the line of the central sulcus. This orientation elicits the lowest threshold MEPs with a latency that is approximately 1–2 ms longer than the MEPs evoked by direct electrical stimulation of corticospinal axons (Day et al., 1989). If the direction of current is reversed (anterior–posterior, AP), threshold is higher and the MEP onset is 2–3 ms later (Day et al., 1989). Recordings from the cervical cord of human patients with implanted epidural electrodes show that PA stimulation evokes highly synchronized corticospinal (CS) activity, whereas AP stimulation preferentially evokes less synchronized, and delayed CS activity (Di Lazzaro and Ziemann, 2013). These outputs have been assumed to result from activity in independent circuits that have different synaptic input pathways to CSNs (Di Lazzaro and Ziemann, 2013). We will refer to them as PA-sensitive and AP-sensitive inputs. Recent papers suggest that these sets of inputs may respond differently to synaptic plasticity protocols (Koch et al., 2013), short-interval intracortical facilitation between two closely spaced TMS pulses (Delvendahl et al., 2014), and short-latency afferent inhibition between a peripheral nerve stimulus and TMS pulse (Ni et al., 2011).

The first aim of the present study was to test whether the S–D time constants of these inputs differ, thus providing further evidence that they represent activity in two separate pathways. The second, related aim was to try to improve the selectivity of AP stimulation. The reason is that the onset latency of MEPs evoked by AP stimulation, relative to direct activation of the corticospinal axons, varies considerably between individuals. In contrast, latencies to PA stimulation are relatively similar. Hamada et al. (2013) have suggested that this is because AP stimulation, which has a higher threshold than PA stimulation, recruits a mixture of “pure” (late onset) AP-sensitive inputs to corticospinal neurons plus a proportion of (early onset) PA-sensitive inputs. If the probability that an AP pulse activates the PA-sensitive inputs varies from person to person, perhaps because of subtle differences in anatomy or physiology of individual brains, it would explain the variation in MEP onset latencies that is observed. We reasoned that it might be possible to tailor the duration of TMS pulses to recruit AP-sensitive inputs more selectively. The third aim was to compare these estimates of central motor S–D time constants with those measured using the same TMS techniques on peripheral motor axons in the ulnar nerve, in order to illustrate that measurements in the cortex reflect the membrane properties of large myelinated axons.

## 2 Material and methods

### 2.1 Subjects

Subjects for the main experiment involving cortical stimulation with TMS were ten healthy, right-handed volunteers (5 women, 5 men; mean age = 29 ± 5 years) with no history of neurological, psychiatric or other medical problems. Twelve healthy subjects (3 women 9 men; mean age 29 ± 7 years; 10 right-handed, 1 left-handed, 1 ambidextrous) participated in the control experiment involving peripheral magnetic stimulation of the ulnar nerve. The study was approved by the Ethics Committee of the University College London.

### 2.2 cTMS device

**Fig. 1:**
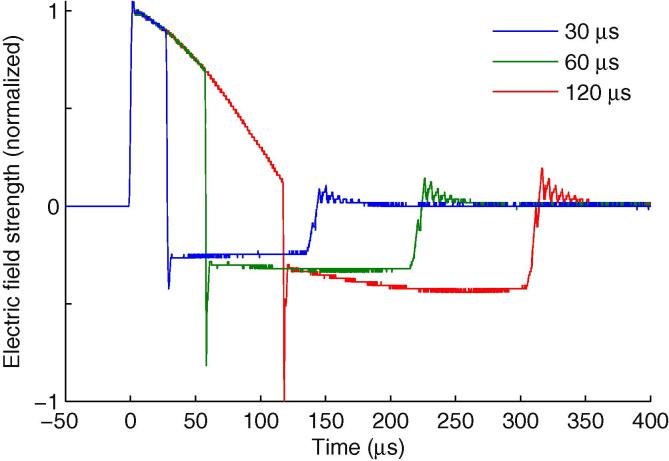
cTMS electric field pulse waveforms recorded with a search coil and normalized to unity amplitude for pulse widths of 30, 60 and 120 μs. Reproduced from Peterchev et al. (2014).

Single pulse stimulation was performed using a custom built cTMS device (cTMS3) that generates triangular monophasic magnetic pulses that induced near-rectangular electric field pulses with independent control of the pulse width (Peterchev et al., 2014), connected to a standard figure-of-eight coil with an wing diameter of 70 mm (The Magstim Co. Ltd.). Like conventional monophasic pulses, the electric field pulse consists of a main phase, followed by a lower amplitude but longer lasting phase in the opposite direction (Fig. 1). Like all TMS pulses, the two phases have equal area under the curve, and hence no net electrical charge is delivered to the tissue. The pulse width refers to the duration of the first, dominant phase of the electric field pulse. Three pulse widths were used in this study for studying motor cortex responses (30, 60 and 120 μs) and four for examining peripheral motor axon responses (30, 60, 90 and 120 μs). Each electric field pulse waveform delivered by the cTMS was recorded with a search coil (Fig. 1) and used in the S–D time constant analysis. The pulse amplitude was limited by the cTMS device to 100, 55, 42, and 37 percent of maximum amplitude (% MA) at pulse widths of 30, 60, 90, and 120 μs, respectively. For reference, 100% MA of this cTMS device corresponds to 95% MA of the Magstim 200^2^. More detailed discussion of the cTMS device output and comparison with other devices is provided by Peterchev et al. (2014).

### 2.3 EMG recording

EMG activity was recorded from the right first dorsal interosseous (FDI) muscle via Ag/AgCl cup electrodes with the active electrode placed over the motor point and the reference on the metacarpophalangeal joint of the index finger. The ground electrode was placed over the right wrist. Responses were amplified with a gain of 1000, filtered with a bandpass filter of 20–2000 Hz. Signals were digitized at 5 kHz (Digitimer, Welwyn Garden City, United Kingdom) and recorded by a computer with the use of Signal 5 (Cambridge Electronic Design, Cambridge, United Kingdom).

### 2.4 Procedure

Subjects were seated on a comfortable chair. The coil was placed tangentially to the scalp over the left (dominant) hemisphere with the handle pointing backwards and laterally at 45° from midline, approximately perpendicular to the central sulcus, inducing a PA current in the brain. The motor hot spot, defined as the larger MEP in the contralateral muscle with the lowest intensity, was determined by moving the coil in ∼0.5 cm steps around the hand motor area. The motor hot spot was checked for AP coil orientation as well. There was no difference in the hot spot between PA and AP coil orientation, as has been reported previously (Arai et al., 2005, Jung et al., 2012, Sakai et al., 1997). The position was marked with a red pen on an elastic cap placed on the subject’s head. The coil was always maintained in the same position during each condition of the experiment.

The resting motor threshold (RMT) and active motor threshold (AMT), defined as the lowest stimulation intensity that evoked MEPs >50 μV and >200 μV respectively in at least 5 out of 10 consecutive trials, was determined at the hot spot for each pulse width (30, 60, 120 μs) and coil orientation (PA and AP). For AMT measurement, subjects were asked to maintain approximately 10% contraction of the muscle. Three pulse widths were used for investigating motor cortical responses compared to four for peripheral motor axons (see below), because three pulses has been shown to be sufficient for estimating cortical SD time constants (Peterchev et al., 2013) and because the experimental sessions we already long with many conditions, thus we were keen to avoid the potential for discomfort and fatigue of participants.

IO curves were obtained for each pulse width and orientation at rest and in active contraction. MEPs were recorded starting from the minimum to the maximum intensity, in steps of 2% output. The minimum and maximum number of distinct pulse amplitudes per subject and condition were 14 and 45 respectively, depending of the pulse width. The maximum intensity was determined either by the maximum the subject can tolerate or by the maximum that can be reached by the device. The relationship between intensity and the MEP size was fitted by a cumulative Gaussian Sigmoid function after log-transformation (Peterchev et al., 2013).

At the end of the active contraction condition, 15 additional MEPs were recorded during contraction at 110% AMT for each pulse width and orientation in order to measure the onset latency of MEPs.

### 2.5 Onset latency MEP measurements during active contraction

**Fig. 2:**
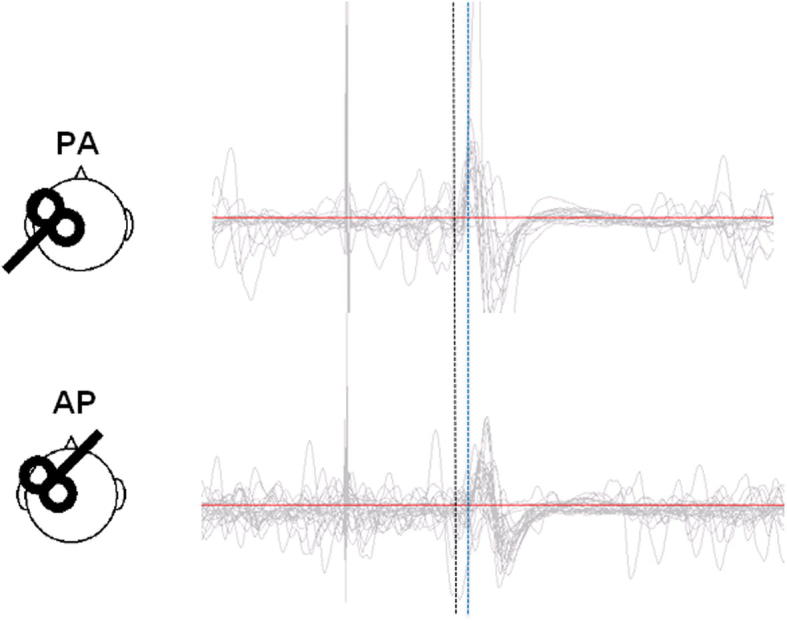
Schematic representation of the coil orientations and typical example of MEPs during contraction by each stimulus for a single pulse width. Dashed line indicates the approximate onset of PA (black line) MEPs for a single pulse width, which is clearly earlier (1–2 ms) than the onset of AP (blue line) MEPs. (For interpretation of the references to color in this figure legend, the reader is referred to the web version of this article.)

Fig. 2 shows representative EMG responses for PA and AP coil orientation in a single subject. Latencies are usually longer for AP than PA orientation (Sakai et al., 1997). Indeed, PA orientation preferentially elicits early I-waves whereas AP orientation recruits late I-waves (Di Lazzaro et al., 2001). Here, the onset latency was measured in a similar way to previous research (Hamada et al., 2013). Two methods were used. First, the onset latency was detected from superimposed waveforms by visual inspection (Di Lazzaro et al., 2001, Rothwell et al., 1987, Shirota et al., 2011). Second, the onset latency was measured trial by trial using previously adopted criteria, whereby the onset was defined as the time point where rectified EMG signals exceed an average plus two standard deviations of the pre-stimulus EMG level (−100 to 0 ms of TMS) (Hamada et al., 2013). These latencies were then averaged for each subject and coil orientation.

### 2.6 Peripheral motor axon stimulation with cTMS

Ulnar stimulation with various pulse durations allowed determination of the strength–duration time constant of peripheral motor axons for comparison with values obtained in the motor cortex. EMG activity was recorded from the FDI at rest using the same recording system as described earlier. Subjects lay supine on a comfortable treatment table with their arms extended by their side. The coil was placed distally on the posteromedial aspect of the upper arm on the dominant side, with the handle parallel to the presumed orientation of the ulnar nerve and the induced current oriented proximal–distal. The optimal position was defined as that producing the largest compound muscle action potential (M-wave) amplitude for a fixed submaximal stimulator intensity with a 120 μs pulse.

IO curves were measured for four pulse widths (30, 60, 90 and 120 μs) in a random order at rest. Stimulus intensity was increased from the minimum to maximum intensity in steps of 1% (90 and 120 μs) or 2% (30 and 60 μs) of maximum stimulator output, giving a minimum and maximum number of distinct pulse steps of 24 and 46, respectively. The maximum stimulus intensity was determined by the maximum possible with the device for each pulse width.

### 2.7 Recruitment curve model

From the recruitment curves, we extracted parameters using least-squares curve regression. Curves were fitted in a mixed model in the log domain (Goetz et al., 2014, Goetz and Peterchev, 2012, Nielsen, 1996, Peterchev et al., 2013). The model was a Gaussian-type curve with four parameters:

(1) $$y=y_{l}+(y_{h}-y_{l})\cdot \Phi \left(\frac{x-m}{s}\right)=y_{l}+(y_{h}-y_{l})\cdot \Phi \left(\frac{x-m}{m\cdot s_{n}}\right)$$

where *x* is the stimulation strength, *y* is the log-transformed peak-to-peak response amplitude, *y_l_* is the low-side plateau, *y_h_* is the high-side plateau (also referred to as saturation level), *m* is the midpoint, *s* is the stretch, *s_n_* = *s*/*m* is the normalized stretch, and *Φ* is the cumulative Gaussian function. Since the many conditions allowed only a limited number of samples per curve, the degrees of freedom of above regression model were successively reduced in three models.

Each of these models had a different abstraction level to find the best balance between over-fitting on the one hand and a high model-determined bias on the other. The former is usually associated with spurious or divergent parameters that cause a high-variability regression; the latter is a result of insufficient flexibility of the model. Instead of incrementally fixing more and more parameters to given values, the degrees of freedom were reduced by sharing parameter values whose difference is lower than the variability of the data across closely related conditions so that they are still individual to a subject, but for instance no longer on other stimulation conditions. We designed a set of three related models, in which the way of reduction was informed by statistically insignificant dependencies. Despite the *F*-test-based design of the models, we independently identified the most appropriate one using Bayesian model selection (Guyon et al., 2010). Such multilevel modeling approaches are recommended for repeated measures (Goldstein, 2011, Mehta and Neale, 2005) and known to perform well when a dataset’s intraclass correlation is high (Roberts, 2004). The more common alternative of fixing parameters, on the other hand, is less flexible and risks spurious results.

For cortical stimulation, the first model performed an individual regression of every recruitment curve condition (2 current directions × 2 muscle activation levels × 3 pulse widths = 12 conditions per subject), leading to 480 degrees of freedom in total. The number of samples was 2740. In this model, the saturation level, *y_h_*, and the normalized stretch, *s_n_*, did not significantly depend on the current direction (*F*(1, 1) = 2.4068, *p* = 0.1238 for *y_h_*; *F*(1, 1) = 2.0611, *p* = 0.1541 for *s_n_*) or the pulse width (*F*(1, 1) = 2.1382, *p* = 0.1230 for *y_h_*; *F*(1, 1) = 0.9993, *p* = 0.3716 for *s_n_*). Therefore, in the second model, the saturation level, *y_h_*, was assumed to depend on the subject and the muscle activation level but not on the current direction and the pulse width, reducing the degrees of freedom to 380. In the third model, the normalized stretch *s_n_* was also made independent of the current direction and the pulse width (Peterchev et al., 2013) so that it is only a parameter of subject and muscle activation level, reducing the degrees of freedom to 280. For a statistical model selection among the three abstraction levels, we evaluated Schwarz’ Bayesian information criterion for every one based on the recorded recruitment curve data (Schwarz, 1978). Schwarz’ information criterion identifies the best fitting model (corresponding to lowest criterion score), mediating between over-fitting with too many degrees of freedom in combination with the underlying dataset and inaccuracy by too few degrees of freedom, and was designed particularly for small sample sizes (Schwarz, 1978). In the context here, it determines which level of abstraction either improves the predictive quality or introduces a bias. Schwarz’ Bayesian information criterion scores were 3870, 3080, and 2301 for Model 1, 2, and 3, respectively. Therefore, Model 3 with the lowest information, i.e. asymptotically with the lowest amount of patterns in the residuals, was used to fit the cortical IO data. The pairwise differences of the Bayesian information scores of the models approximate twice the natural logarithms of their Bayes factor, which in turn quantifies the posterior odds that one model is more appropriate than the other one (Jeffreys, 2003, Kass and Raftery, 1995). A difference of more than 700 for the third model compared to others shows decisive weight of evidence for Model 3 being the appropriate model, in which the goodness of fit is worth the necessary number of variables according to Bayesian statistics (Good, 1992, Kass and Raftery, 1995).

For the stimulation of the peripheral ulnar nerve, model two with the saturation level being independent from the current direction and the pulse width processed the four conditions per subject (4 pulse widths).

### 2.8 Strength–duration time constant estimation

We used the same time-constant estimation procedure as in (Peterchev et al., 2013). The relationship between the motor threshold and the pulse width is usually described by a strength–duration curve parameterized by time constant and rheobase. The former is a measure of excitability of the nodal membrane, derived from the relationship between the strength and duration of the pulse required to elicit an action potential whereas the latter is the threshold for an infinitely long pulse (Bostock and Rothwell, 1997, Mogyoros et al., 1999).

The strength–duration time constant depends on the local electric field distribution and the biophysical properties of the axonal membrane. The MT is dependent on the pulse width and can be modeled by the following equation that represents the strength–duration curve model:

(2) $$V_{\mathrm{th}}^{\prime }(t_{p})=\frac{V_{\mathrm{th}\infty }}{r(\tau_{m}\text{,}t_{p})}$$

where $V_{\mathrm{th}}^{\prime }(t_{p})$ is the modeled MT, *V*_th∞_ is the rheobase, *t_p_* is the pulse width, $\tau_{m}$ is the time constant, and *r*(*τ*_m_, *t_p_*) is the depolarization factor (Peterchev et al., 2013). To estimate the rheobase and time constant, the empirical MT data, $V_{\mathrm{th}}(t_{p})$, were fitted to the parametric model $V_{\mathrm{th}}^{\prime }(t_{p})$ by minimizing the sum of ${\left(\frac{V_{\mathrm{th}}^{\prime }(t_{p})}{V_{\mathrm{th}}(t_{p})}-1\right)}^{2}$ across all pulse widths. For more details, see Peterchev et al. (2013).

In addition, we estimated the S–D time constants from the recruitment curve data for the four stimulation conditions formed by the combination of current direction and muscle activation level in the motor cortex for each subject. The threshold values *t* and the midpoints *m* of the recruitment curves are accordingly given by:

(3) $$t=\frac{t_{0}}{r(\tau_{m}\text{,}t_{p})}\;\text{and}$$

(4) $$m=\frac{m_{0}}{r(\tau_{m}\text{,}t_{p})}$$

The linear S–D time constant *τ*_m_ and the constants *t*_0_ and *m*_0_ were evaluated with a least-squares regression.

Finally, we estimated the S–D time constant for peripheral stimulation of the ulnar nerve as well using the same approach.

### 2.9 Statistical analysis

Two-way repeated measures ANOVA was used to evaluate the influence of coil orientation (AP, PA) and pulse width (30, 60, 120 μs) on MEP onset latencies. Three-way repeated measures ANOVA was used to evaluate the influence of coil orientation (AP, PA), pulse width (30, 60, 120 μs) and muscle activity (rest, active) on motor thresholds and the slope of the IO curves. The Greenhouse–Geisser correction was applied where necessary to correct for violations of sphericity. Paired-samples *t*-tests were used to compare cortical S–D time constants between coil orientations, using the individualized data, for the resting and active conditions.

## 3 Results

### 3.1 Motor cortex latency differences

**Fig. 3:**
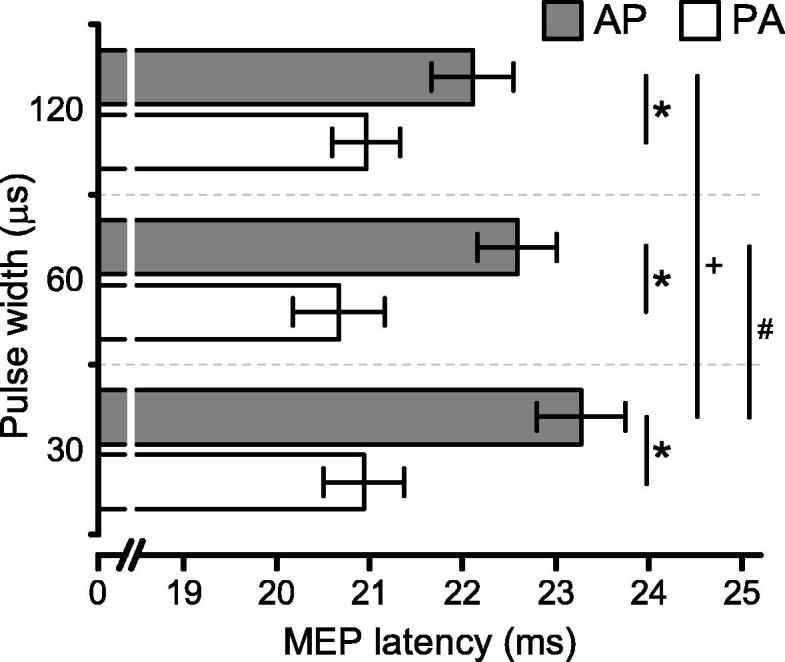
Onset latencies of MEPs recorded during slight contraction with PA- and AP-directed currents, each with 30, 60 and 120 μs pulse widths. There was a significant interaction of pulse orientation and pulse duration (*p* = 0.001) and a simple effect of the pulse duration for AP condition (*p* = 0.01). Data are mean ± SEM. * indicates *p* < 0.001 for PA versus AP, + indicates *p* < 0.001 for AP 30 μs versus AP 120 μs, # indicates *p* < 0.05 for AP 30 μs versus AP 60 μs.

Repeated measures ANOVA on MEP latencies to AP and PA stimulation (during active contraction) revealed a main effect of orientation (*F*(1, 9) = 65.12; *p* < 0.001): as expected the MEPs had an earlier onset after PA than AP stimulation. In addition there was a significant interaction between pulse width and orientation (*F*(2, 18) = 9.97; *p* = 0.001), due to an increase in onset latencies with decreasing pulse duration for AP but not PA stimuli (AP 30 = 23.3 ± 1.5 ms; AP 60 = 22.6 ± 1.34 ms; AP 120 = 22.1 ± 1.4 ms) (Fig. 3).

### 3.2 Motor cortex S–D curves

**Fig. 4:**
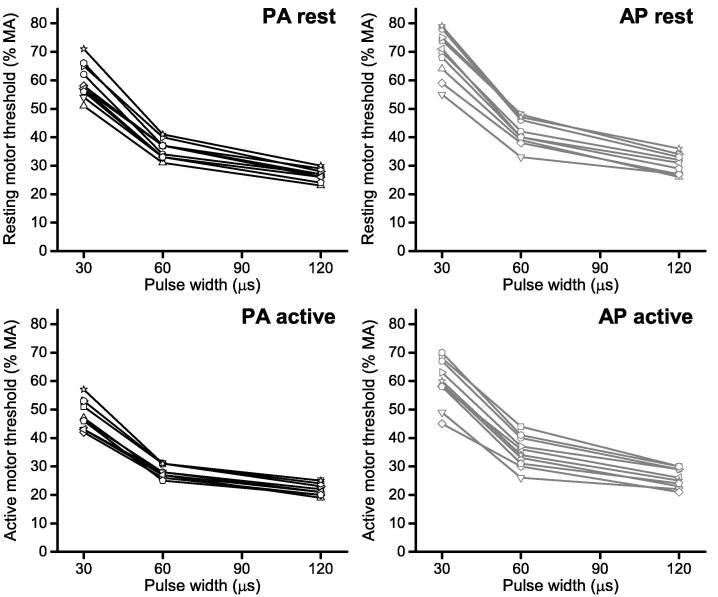
Strength–duration curves shown for each coil orientation at rest and during slight contraction, where stimulus strength reflects motor threshold (MT) as % maximum pulse amplitude (% MA) and duration is the pulse width (μs). Data are shown for individual participants (*n* = 10).

Strength–duration curves (i.e. MT threshold as a function of pulse width for each subject) are shown in Fig. 4. Curve parameters were estimated for each subject using the neural response model. Results are summarized in Table 1. First, parameters were estimated for each subject under the assumption of individual time constant and rheobase (20 parameters for each of the 4 conditions). Second, we assumed an individual rheobase but a common time constant for all subjects (11 parameters per each of the 4 conditions).

Using the individualized estimates from the first model, repeated measure ANOVA on S–D values showed a main effect of coil orientation (*F*(1, 9) = 39.20; *p* < 0.001), pulse width (*F*(1, 9) = 633.06; *p* < 0.001) and contraction (*F*(1, 9) = 96.31; *p* < 0.001), as well as an interaction between contraction and pulse width (*F*(2, 18) = 29.56; *p* < 0.001) and between orientation and pulse width (*F*(2, 18) = 29.77; *p* < 0.001). The 3-way interaction approached significance (*p* = 0.076). The mean time constant was significantly longer in AP than PA orientation during a weak background contraction (*t*(9) = 3.39; *p* = 0.008), although there was no difference between orientations at rest (*t*(9) = 0.51 ; *p* = 0.62).

### 3.3 Motor cortex IO curves

**Fig. 5:**
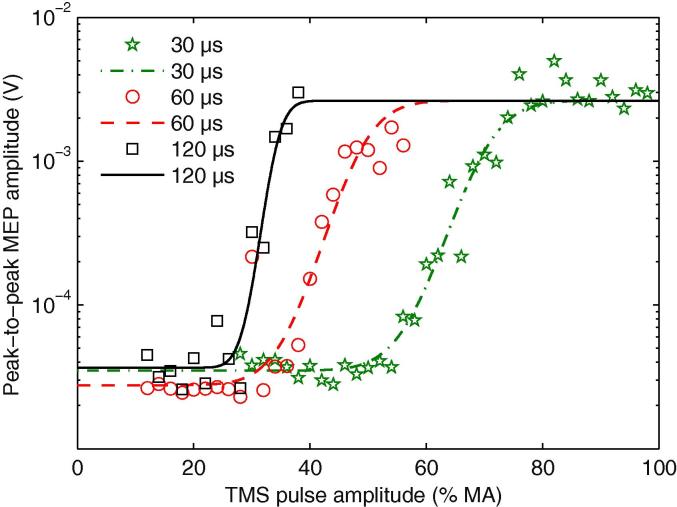
Example IO curves of one subject for PA stimulation at rest with pulse widths of 30, 60 and 120 μs. The markers show measured data points (peak-to-peak amplitude of an individual MEP) as a function of the TMS pulse amplitude. The lines show the best-fitting cumulative Gaussian model. In this model, identified by Bayesian model selection, all three pulse widths share the saturation level, which is individual but independent from the pulse width. For equal peak voltage amplitude of the pulses, longer pulses have a lower threshold so that the IO curves of 120 μs and 60 μs pulses are shifted to the left towards lower stimulation strengths. Note the logarithmic scale of the vertical axis, which renders the MEP variability more normal.

The most complete dataset was obtained from individuals during active contraction since limitations of the stimulator output meant that in some participants we were unable to reach a plateau value for MEP amplitude when excitability was reduced at rest. Repeated measures ANOVA on the slope showed an influence of the three factors: pulse width (*F*(1, 9) = 60.79; *p* < 0.001), orientation (*F*(1, 9) = 21.87; *p* = 0.001), and contraction (*F*(1, 9) = 23.48; *p* < 0.001), characterized by an increased slope for longer pulse width, PA orientation and during contraction. Additionally, the ANOVA was significant for the interaction between pulse width and orientation (*F*(2, 18) = 3.83; *p* = 0.04), pulse width and contraction (*F*(2, 18) = 9.18; *p* = 0.001), and contraction and orientation (*F*(2, 18) = 6.77; *p* < 0.028). A representative set of curves for each pulse width at rest during PA oriented current stimulation from one subject are displayed in Fig. 5.

S–D time constants were also estimated from IO data. The resting time constants were more complicated to extract because of variability in the data. However, during active contraction, time constant estimates from IO data were close to those from MT data. Indeed, we estimated a time constant derived from IO data of 251 and 295 μs for PA and AP orientation respectively (*t*(9) = 8.7, *p* < 0.001). Estimates of time constants at rest were considered unreliable since plateau MEP amplitudes could not be obtained with this TMS device in many individuals that we tested (see above).

### 3.4 S–D time constants of ulnar nerve motor axons

The S–D time constant estimated from the IO curves of motor axons of the ulnar nerve was 197 ± 47 μs, and was therefore close to those obtained in the motor cortex.

## 4 Discussion

The present results show that, when measured in actively contracting muscle, the S–D time constant for threshold stimulation of M1 is longer for AP than PA pulses; both lie within the range of estimates of ulnar nerve motor axon time constants calculated with the same methods. The difference in values for AP- and PA-pulses is compatible with the idea that the two directions of current activate different populations of inputs to the corticospinal neurons, or that they activate the same populations at different sites. A further novel finding was that AP stimulation with narrow (30 μs) pulses evoked MEPs with the longest onset latency. The possible reasons for this are discussed below.

### 4.1 S–D time constant

The S–D time constant is a commonly applied measure of the excitability of an axon. It is a measure of how the duration of a threshold stimulus pulse varies as a function of its amplitude. This depends on the magnitude of persistent Na+ currents, membrane potential and the passive electrical properties of the membrane. It is equivalent to chronaxie, the stimulus duration of a pulse at twice rheobasic strength (rheobase is the minimum amplitude of an infinitely long stimulus pulse).

Interpretation of the S–D time constant in the present experiments is more complex than for stimulation of a peripheral nerve since a number of synapses are interposed between the site of activation in cortex and the EMG response recorded from muscle. For example populations of cortical axons activated by TMS could have synaptic relays with different probabilities of transmission. They could recruit the same population of corticospinal neurones or separate subpopulations, conceivably with projections to subpopulations of spinal motoneurones. We tried to minimize the mixture of cortical elements activated by the TMS pulse by measuring the S–D time constant for just-suprathreshold stimuli since they should tend to activate only the most excitable elements. Nevertheless, we cannot exclude the possibility that the S–D time constant could be a composite from more than one axonal population. For example, the lowest threshold axons could have relatively inexcitable synaptic connections that alone were incapable of exciting corticospinal output. Only when supplemented by excitation from synapses activated by higher threshold axons would an output be produced. In this case, the S–D time constant would reflect activation of two sets of axons even at threshold. The fact that we obtained different estimates of S–D time constant for PA and AP stimulation suggests that they activate at least partially separate sets of cortical axons.

The possibility that AP and PA stimulation activate the same axons at different sites (perhaps due to differences in current direction relative to the axon trajectory) seems less likely given the different characteristics of the I-waves generated by AP and PA pulses. As noted in the Introduction, I-waves produced by AP stimulation are not simply a time-delayed reproduction of the I-waves evoked by PA stimulation, but differ in their latencies and variability (Di Lazzaro and Ziemann, 2013).

The cTMS device that we used could change the stimulus duration over a fourfold range from 30 to 120 μs, which is short compared with the usual range of durations (often up to 1 ms) used to estimate conventional S–D constants with electrical stimulation (Mogyoros et al., 1999). Because of the smaller range of values, two methods were used to extract the time constant: first, a strength–duration curve model using MT values and second, a recruitment curve regression using IO data. The latter can be achieved because the slope of the IO curve is steeper for longer pulse widths. This can be explained by the same membrane depolarization mechanisms that shape the MT strength–duration relationship (Peterchev et al., 2013). We found a longer time constant for AP than PA orientation whatever the method used for estimation. Our time constant values for PA orientation (around 250 μs) are similar to a previous cTMS study that estimated a time constant at rest of 196 μs from MT data (Peterchev et al., 2013), which is included in our 95% confidence interval [190–295 μs]. They are both slightly higher than the 152 ± 26 μs estimated for cortical stimulation by Barker et al. (1991), although the latter only used a simple strength–duration model to estimate time constants rather than the more complex curve fitting approach taken here. Despite this, the values for central S–D time constants of both the present data and that of Barker et al. (1991) are very similar to the values for activation of peripheral motor axons as estimated with the same methods, suggesting that TMS of motor cortex activates myelinated axons of large diameter. If TMS were activating small or non-myelinated axons, one would have expected cortical time constants to be greater than peripheral motor axon time constants by a factor or two or more (West and Wolstencroft, 1983); however, the difference in the present data was less than 50%. Thus it seems likely PA- and AP-sensitive axons are within the range of large myelinated axons.

With the coil orientation we used here TMS is not thought to activate directly the large axons of corticospinal neurones. Instead, it activates other fibers that have synaptic connections with the corticospinal neurones (Day et al., 1989). Liewald et al. (2014) recently measured the diameter of axons in three different subcortical fiber tracts and found values up to 9 mm in post mortem human material. These would be expected to have conduction velocities of up to 40–50 ms^−1^ (Hursh, 1939). Presumably such fibers would be a prime target for activation with TMS, perhaps as their axons enter the cortical gray matter (Thielscher et al., 2011).

The S–D time constants estimated with here with TMS are shorter than values estimated by conventional electrical stimulation of the median nerve at the wrist (around 400 μs). They are also shorter than the electrical S–D time constant of corticospinal axons estimated intraoperatively by Burke et al. of 432 μs (Burke et al., 2000). One possible reason for the difference is that simulation studies have shown a strong dependence of the time constant on the electrode distance for implantable electric stimulation, ranging between 220 μs and 574 μs for the same human motor axon (Kuhn et al., 2009). The equivalent values for TMS and conventional electrical stimulation could differ considerably. Two further factors could contribute to the difference between our TMS data and the higher electrical time-constant values from the literature. First, the shapes of the cTMS pulses are not perfectly rectangular, like those from a conventional electric stimulator, since the intensity declines throughout the pulse. In addition they have a reverse phase that prevents net charge deposition in the tissue. The second factor is the relatively small range of pulse durations that can practically be used with TMS devices (up to 120 μs in the present experiments, compared with >1000 μs for electrical stimulation). For longer durations of magnetic pulses both the pulse energy and the losses, i.e. coil heating, increase above feasible limits (Goetz et al., 2013). Thus, with existing devices, TMS cannot probe the effects of long duration pulses. This influences particularly the estimation of neural parameters such as the rheobase, which is defined as the stimulation threshold for infinitely long pulses. Here, the rheobase is estimated by regression from the available short pulses, leading to a potentially high uncertainty of the estimation.

The S–D time constant of AP stimulation was significantly longer than for PA stimulation when estimated during active contraction whereas it did not differ when participants were at rest. This is probably explained by physiological differences in the recruitment of MEPs at rest as compared with the preactivated state. Resting motor threshold is higher than active threshold because more descending activity is required to raise resting spinal motor neurons to threshold than when they are active. Importantly, resting threshold for AP stimulation is higher than for PA stimulation. This means that when AP stimulation is used to evoke an MEP at rest, the stimulus intensity is large compared with the threshold for PA stimulation. Thus when finding threshold at rest, AP stimulation may first recruit cortical “AP-neurones” but this may be insufficient to discharge quiescent spinal motoneurones. As the intensity (or pulse width) is increased, it may become high enough to recruit some PA-sensitive inputs, which will provide additional corticospinal input to spinal motoneurones and threshold will be reached. In this case, AP stimulation will result in an S–D time constant equal to that of PA stimulation. This would also fit with the usual finding that at rest there is little or no difference in onset latency of MEPs evoked by either direction of stimulation (Di Lazzaro et al., 2001). During activation, spinal motoneurones are more excitable and can probably respond to the initial corticospinal excitation provided by pure AP stimulation. In this case, AP and PA S–D time constants will differ.

### 4.2 MEP recruitment by AP stimulation

As expected, the onset latency of MEPs evoked in actively contracting muscle was 2–3 ms longer with AP than PA stimulation. Day et al. (1989) initially proposed that threshold PA stimulation recruited monosynaptic input to corticospinal neurons, and that when these discharged, an I1-wave was evoked (Amassian et al., 1987) in the corticospinal tract. In contrast, they suggested that threshold AP stimulation recruited later I2 or I3 inputs to the same corticospinal neurons. This appeared to account for the latency differences in surface MEP responses as well as the recruitment of single motor units in the same muscles. However, the situation may be more complex than this. Direct recordings in cervical cord of the descending corticospinal volleys evoked in conscious human patients with implanted epidural electrodes for relief of pain showed that although the latency of the earliest volleys evoked by PA and AP stimulation differed, the timing and shape of the volleys did not match completely. Although PA stimulation evoked clear I waves, those recruited by AP stimulation were more dispersed and their peaks did not necessarily match the timing of the PA-evoked activity (Di Lazzaro et al., 2001). The implication is that the inputs activated by threshold AP and PA stimulation are probably different: those activated by AP stimulation tend to take longer to activate corticospinal output neurons than those activated by PA stimulation. We therefore refer to them as AP-sensitive and PA-sensitive inputs (to corticospinal output neurons), which the present results now show to have different S–D time constants.

A remaining question is why the onset latency of MEPs evoked with AP stimulation varied with pulse duration: the longest latency MEPs were evoked with 30 μs stimuli whereas the latency was 1 ms shorter with pulses lasting 120 μs. One possibility is that an AP oriented pulse activates more than one population of axons (see arguments above). Pulses with a duration of 120 μs may activate axons with a more rapid input to corticospinal neurons than pulses with a duration of 30 μs.

The idea that AP stimulation can activate more than one input to corticospinal neurons is consistent with the results of Di Lazzaro et al. (2001) who noted that the onset latency of the earliest volleys recruited by threshold AP stimulation differed relative to D-wave latency in each of their four participants. They suggested that AP stimulation can recruit a variety of possible inputs to corticospinal neurons that have varying onset latencies and different recruitment thresholds in different people. The source of this variation is unknown, but it probably contributes to the range of MEP onset latencies that can be observed between individuals (Hamada et al., 2013). Our hypothesis is that short duration pulses preferentially activate AP-sensitive inputs that take the longest time to excite corticospinal neurons, and thus produce the longest latency MEPs.

### 4.3 Clinical and research implications

The cTMS device used here permitted a fourfold range of stimulus durations (Peterchev et al., 2014), enabling the estimation of cortical SD time constants. Additionally, MEP latencies of AP-directed stimuli were shown to vary with pulse duration. Direct comparison of the present results with commercially-available devices is difficult since most permit little or no control over pulse width (Peterchev et al., 2014, Rothkegel et al., 2010) and exhibit a sinusoidal pulse shape which differs from the more rectangular pulses delivered via the cTMS device (Peterchev et al., 2011). However, the frequently used Magstim 200^2^ stimulator (The Magstim Company Limited, UK) has a pulse width of 82 μs (Rothkegel et al., 2010), which lies in the upper range of pulse widths examined in the present study (30–120 μs). Based on the present data we might expect latencies for AP and PA stimulation with Magstim 200^2^ to fall within the range observed for 60 and 120 μs pulses, such that difference in latencies for AP-directed stimuli with a Magstim 200^2^ versus a 30 μs pulse with the cTMS device would be expected to be ∼1 ms. Thus the new cTMS device may enable, via the selection of short AP-directed pulses, the potential to more selectively activate specific neural populations (i.e. long latency inputs). At present it is not clear how this might impact the utility of TMS as a research, diagnostic or therapeutic tool, but this is something that will be examined in further experiments. Similarly, the potential to quantify cortical SD time constants may offer novel insights into the influence of pathologies and CNS active drugs on motor cortical axon physiology, but these possibilities remain to be tested.

In summary, our data suggests that PA and AP orientations of TMS activate neural structures with different time constants. These are likely to be relatively large diameter myelinated axons since the S–D time constants are in the range of those estimated for peripheral motor axons using the same methods. Finally, AP-sensitive inputs that recruit the longest latency MEPs are more readily stimulated by short than by long duration pulses.

## Figures and Tables

**Table 1:** Strength–duration curve parameters determined from the MT at rest and during contraction for PA and AP directed stimuli. Data area mean ± SD (*n* = 10).

Model	Parameters	PA rest	AP rest	PA active	AP active
Individual rheobase & time constant	Rheobase (% MA)	6.29 ± 1.05	7.24 ± 1.84	5.83 ± 1.65	5.54 ± 1.32
Time constant (μs)	251 ± 55	268 ± 97	231 ± 97⁎	294 ± 91⁎

Individual rheobase & group time constant	Rheobase (% MA)	6.31 ± 0.55⁎	7.21 ± 0.81⁎	5.78 ± 0.53	5.47 ± 0.75
Time constant (μs)	243	249	210	282

⁎ *P* < 0.01 for comparisons between AP and PA oriented pulses within each contraction condition (i.e. rest or active).
